# Superhuman Strength in Cancer

**DOI:** 10.6004/jadpro.2016.7.6.2

**Published:** 2016-09-01

**Authors:** Laura Melton

**Affiliations:** Dr. Melton is the Medical Director of Supportive Oncology and an Assistant Professor in the Division of Medical Oncology at the University of Colorado.

Sarah was in her mid-30s with widely metastatic breast cancer when I met her. Her sole purpose in life was to be a mother. She worked with high schoolers and thought of herself as an extended mom to some of the students. When fertility issues arose, she sought treatment. In our first session, I said, "Tell me about yourself." Most patients think for a bit about how they want to respond. But without skipping a beat, Sarah replied, "I’m a mom. That’s what I’m here to do."

**The Power of Identity**

Sarah had two children: a daughter and a (deceased) son. Her son had been found to have a life-threatening condition while in the womb, so he was delivered early, with no chance for survival. His ashes rested on the family mantel, the most prominent place in the home. Whenever her daughter would get a balloon at an event (a birthday party, the circus, or the grocery store), Sarah would encourage her to release the balloon into the sky, saying, "Let’s share this balloon with your brother." Sarah wanted to make sure both of her children constantly felt her love.

When discussing her cancer, Sarah would say, "This is not how my story is supposed to end!" It was supposed to end when she was 110, surrounded by her children, her grandchildren, and her great grandchildren. It was not supposed to end in her mid-30s.

One of my colleagues who shared some patients with me as well as a common office wall would see me in the hall later in the day after my session with Sarah and say, "I know who was in your office today. I heard her screaming…I’m glad you’re here to help when she screams because I don’t know what to do." Sarah screamed in my office over and over about how having cancer—especially incurable, fast-growing, nasty cancer—was not how her life was supposed to go.

**Superhuman Endurance**

Sarah’s screaming was common in our sessions because she wanted to stay positive for her family and friends, but after a 3-year-long, grueling battle with cancer, this was hard to do. She was starting to crack. She sometimes snapped at family or friends who asked if they could pick up her daughter from school—something they thought was helpful, but that she took as a sign of weakness and giving up.

One day I saw Sarah in the infusion room instead of my office, our traditional meeting place for therapy. At this point, we knew the chemo was not stopping the cancer because she had lumps sticking up through her skin around her neck area that she could see and feel growing between chemo rounds. Scan after scan showed the metastases continuing to grow, but she kept getting chemo. Her oncologist and I would tag-team the, "It’s OK to think about quality of life instead of quantity of life" talk, but she wanted none of it. She was determined to be a "miracle" and beat her aggressive stage IV breast cancer.

When I saw Sarah in infusion that day, she was exhausted and a ghost of herself. She had to lay down in a bed to get her chemo, unable to expend the energy needed to sit in a reclining chair. She smiled at me and said, "I’m going to beat this," and then had to rest before she could say another sentence. She noted that she was so exhausted from her chemo (coming to the hospital from her home 40 minutes away, getting labs drawn, receiving chemo over the course of several hours, and then going home) that once she got home all she could do was sleep. Her daughter, the one she fought so hard for, was only seeing a shell of her at home because she was expending all of her energy on getting her chemo.

**Identifying the Person, Not the Cancer**

A cancer journey is very personal. In addition to the medical impact of the cancer itself as well as the side effects of treatment, and the obvious practical and emotional impact a cancer diagnosis has on someone, it’s important for us as caregivers to recognize who the person was before their cancer. The individual’s personal history, social history, culture, family beliefs, roles in life, etc., all impact how someone copes with cancer.

Sarah identified herself first and foremost as a mother, and a mother does everything she can to save her children. Mothers have been documented lifting cars that weigh thousands of pounds to rescue their trapped children. This superhuman strength, a display of tremendous force surpassing what is expected to be normal for humans, comes when people are in life-or-death situations. Sarah fit that criterion. She was fighting for her daughter to have an ideal life complete with a mother, a father, and a white picket fence.

This superhuman strength is thought to come from surges of adrenaline, yet relying on ongoing adrenaline surges for the indefinite future does not seem like a sustainable strategy. Sarah’s entire oncology team kept reminding her that she could stop getting chemo. But what we failed to recognize is that while we were trying to be supportive by saying things like, "Focus on your quality of life," we were not recognizing that in Sarah’s mind, we were essentially asking her to put the car down with her baby still trapped underneath, lay her head down, and hold the baby’s hand. This was not something that was possible for Sarah at this point.

Sarah’s history is the key to understanding her relentless cancer fight. Her identity as a mother—her history of doing anything and everything possible for her children, her initial response to me when I asked her to tell me about herself—was so ingrained that she could not think of herself separate from her children, even when she thought about her cancer. There was never a glimpse of what Sarah the woman would want. It was what Sarah the mother needed to do. As a mother myself, I empathize with her. Sitting with the emotions of another human, watching them suffer to try to save their loved ones, is extremely intense, especially because with her advanced and aggressive cancer, there was no path to allow Sarah to prevent her daughter from losing her mother.

**Coming to Terms With Our Limitations**

As providers, we sometimes fall into the same headspace as Sarah. We want so desperately to save our patients from having to suffer. In oncology, we see displays of superhuman strength all the time: clinicians who keep their pagers on 24/7 and hand out their cell phone numbers so their patients can always reach them, seemingly never-ending phone calls to help transition care, or pushing to get this one particular patient added to a clinical trial that technically does not have any more room. It becomes hard to provide an ongoing stream of that deep empathy that makes us good at our job with such a sustained level of adrenaline needed for these superhuman strength behaviors. Emotional exhaustion and burnout are not surprising in oncology due to this intensity.

Eventually, Sarah could no longer sustain her superhuman strength. Her battle with cancer came to a close when she was weak, lying in a hospital bed trying to argue to be transferred to a rehab facility so she could "get stronger" and "get back to having more chemo," all so she could extend her life and try to be around for more of her daughter’s birthdays. In a moment of low adrenaline, she recognized that she would rather be at home with her daughter at her side than in the oncology wing of the hospital which for immunity purposes doesn’t allow small children, especially during flu season. She was exhausted from an extended feat of superhuman strength. She was ready just to be present with her family, knowing she had done all that she could do.

**Final Thoughts**

As oncology providers, we sometimes need to remember this lesson: This may not be how the patient’s story is supposed to end, but in some cases there comes a time when there is nothing left to do. What we can offer is just to be present with our patients. Sometimes we also have to lower the car, place our head on the pavement, and hold their hand.

